# A systematic review to identify areas of enhancements of pandemic simulation models for operational use at provincial and local levels

**DOI:** 10.1186/1471-2458-12-251

**Published:** 2012-03-30

**Authors:** Diana M Prieto, Tapas K Das, Alex A Savachkin, Andres Uribe, Ricardo Izurieta, Sharad Malavade

**Affiliations:** 1 Department of Industrial and Manufacturing Engineering, Western Michigan University, Kalamazoo, MI 49008, USA; 2 Department of Industrial and Management Systems Engineering, University of South Florida, Tampa, FL 33620, USA; 3 Department of Radiation Oncology, University of California - San Diego, La Jolla, CA 92093-0843, USA; 4 College of Public Health, University of South Florida, Tampa, FL 33620, USA

## Abstract

**Background:**

In recent years, computer simulation models have supported development of pandemic influenza preparedness policies. However, U.S. policymakers have raised several *concerns *about the practical use of these models. In this review paper, we examine the extent to which the current literature already addresses these *concerns *and identify means of enhancing the current models for higher operational use.

**Methods:**

We surveyed PubMed and other sources for published research literature on simulation models for influenza pandemic preparedness. We identified 23 models published between 1990 and 2010 that consider single-region (e.g., country, province, city) outbreaks and multi-pronged mitigation strategies. We developed a plan for examination of the literature based on the concerns raised by the policymakers.

**Results:**

While examining the concerns about the adequacy and validity of data, we found that though the epidemiological data supporting the models appears to be adequate, it should be validated through as many updates as possible during an outbreak. Demographical data must improve its interfaces for access, retrieval, and translation into model parameters. Regarding the concern about credibility and validity of modeling assumptions, we found that the models often simplify reality to reduce computational burden. Such simplifications may be permissible if they do not interfere with the performance assessment of the mitigation strategies. We also agreed with the concern that social behavior is inadequately represented in pandemic influenza models. Our review showed that the models consider only a few social-behavioral aspects including contact rates, withdrawal from work or school due to symptoms appearance or to care for sick relatives, and compliance to social distancing, vaccination, and antiviral prophylaxis. The concern about the degree of accessibility of the models is palpable, since we found three models that are currently accessible by the public while other models are seeking public accessibility. Policymakers would prefer models scalable to any population size that can be downloadable and operable in personal computers. But scaling models to larger populations would often require computational needs that cannot be handled with personal computers and laptops. As a limitation, we state that some existing models could not be included in our review due to their limited available documentation discussing the choice of relevant parameter values.

**Conclusions:**

To adequately address the concerns of the policymakers, we need continuing model enhancements in critical areas including: updating of epidemiological data during a pandemic, smooth handling of large demographical databases, incorporation of a broader spectrum of social-behavioral aspects, updating information for contact patterns, adaptation of recent methodologies for collecting human mobility data, and improvement of computational efficiency and accessibility.

## Background

The ability of computer simulation models to "better frame problems and opportunities, integrate data sources, quantify the impact of specific events or outcomes, and improve multi-stakeholder decision making," has motivated their use in public health preparedness (PHP) [1]. In 2006, one such initiative was the creation of the Preparedness Modeling Unit by the Centers for Disease Control and Prevention (CDC) in the U.S. The purpose of this unit is to coordinate, develop, and promote "problem-appropriate and data-centric" computer models that substantiate PHP decision making [2].

Of the existing computer simulation models addressing PHP, those focused on disease spread and mitigation of pandemic influenza (PI) have been recognized by the public health officials as useful decision support tools for preparedness planning [1]. In recent years, computer simulation models were used by the Centers for Disease Control and Prevention (CDC), Department of Health and Human Services (HHS), and other federal agencies to formulate the "U.S. Community Containment Guidance for Pandemic Influenza" [3].

Although the potential of the exiting PI models is well acknowledged, it is perceived that the models are not yet usable by the state and local public health practitioners for operational decision making [1,4-6]. To identify the challenges associated with the practical implementation of the PI models, the National Network of Public Health Institutes, at the request of CDC, conducted a national survey of the practitioners [1]. The challenges identified by the survey are summarized in Table 1.

**Table 1:** A summary of the survey results on the challenges of practical use of PI models, as perceived by public health practitioners

Challenge	Description of the challenge
**A1. Validity of data support**	Model parameters need to be derived from updated demographical and epidemiological data

**A2. Credibility and validity of assumptions**	Models need to use credible and valid assumptions

**A3. Represent human behavior**	Models need to incorporate human behavior

**A4. Accessibility**	Models need to be easily accessible and run on personal computers

**A5. Scalability**	Models need to be scalable to population specific data from regions of all sizes

**A6. Awareness**	Available models and best practices need to be disseminated among the practitioners

**A7. Action plan**	Need to translate models into uniform preparedness and response action plans

**A8. Lack of resources**	Need to fund staff allocation and specialized training for model implementation

**A9. Political implications**	Models need to consider second and third tier social implications of containment strategies

**A10. Lack of mandates for models**	State and federal agencies need to develop mandates for use of model-based strategies

We divided the challenges (labeled A1 through A10 in Table 1) into two categories: those (A1 through A5) that are related to model design and implementation and can potentially be addressed by adaptation of the existing models and their supporting databases, and those (A6 through A10) that are related to resource and policy issues, and can only be addressed by changing public health resource management approaches and enforcing new policies. Although it is important to address the challenges A6 through A10, we consider this a prerogative of the public health administrators. Hence, the challenges A6 to A10 will not be discussed in this paper.

The challenges A1 through A5 reflect the perspectives of the public health officials, the end users of the PI models, on the practical usability of the existing PI models and databases in supporting decision making. Addressing these challenges would require a broad set of enhancements to the existing PI models and associated databases, which have not been fully attempted in the literature. In this paper, we conduct a review of the PI mitigation models available in the published research literature with an objective of answering the question: "how to enhance the pandemic simulation models and the associated databases for operational use at provincial and local levels?" We believe that our review accomplishes its objective in two steps. First, it exposes the differences between the perspectives of the public health practitioners and the developers of models and databases on the required model capabilities. Second, it derives recommendations for enhancing practical usability of the PI models and the associated databases.

## Methods

In this section, we describe each of the design and implementation challenges of the existing PI models (A1-A5) and present our methods to examine the challenges in the research literature. In addition, we present our paper screening and parameter selection criteria.

### Design and implementation challenges of pandemic models and databases

#### Validity of data support (A1)

Public health policy makers advocate that the model parameters be derived from up to date demographical and epidemiological data during an outbreak [1]. In this paper we examine some of the key aspects of data support, such as data availability, data access, data retrieval, and data translation.

To ensure data availability, a process must be in place for collection and archival of both demographical and epidemiological data during an outbreak. The data must be temporally consistent, i.e., it must represent the actual state of the outbreak. In the United States and other few countries, availability of temporally consistent demographical data is currently supported by governmental databases including the decennial census and the national household travel survey [7-10]. To ensure temporal consistency of epidemiological data, the Institute of Medicine (IOM) has recommended enhancing the data collection protocols to support real-time decision making [4]. The frequency of data updating may vary based on the decision objective of the model (e.g., outbreak detection, outbreak surveillance, and initiation and scope of interventions). As noted by Fay-Wolfe, the timeliness of a decision is as important as its correctness [11], and there should be a balance between the cost of data updating and the marginal benefits of the model driven decisions.

Archival of data must allow expedited *access *for model developers and users. In addition, mechanisms should be available for manual or automatic retrieval of data and its translation into model parameter values in a timely manner.

In our review of the existing PI models at provincial and local levels, we examined the validity of data that was used in supporting major model parameters. The major model parameters include: The reproduction number, defined as the number of secondary infections that arise from a typical primary case [12]; the proportion of the population who become infected, also called infection attack rate [13]; the disease natural history within an individual; and fractions of symptomatic and asymptomatic individuals. The first row of Table 2 summarizes our approach to examine data validity. For each reviewed PI model, and, for each of the major model parameters, we examined the source and the age of data used (A1a, A1b), the type of interface used for data access and retrieval (A1c), and the technique used for translating data into the parameter values (A1d).

**Table 2:** Plan for examination of the design and implementation challenges of the existing PI models

Design and implementation challenges	Plan of examination
Validity of data support (A1) for model parameters	For each PI model and for each of the major model parameters (e.g., reproduction number, illness attack rate) examine:
	A1a. Data source for parameter values (actual, simulated, assumed)
	A1b. Age of data
	A1c. Type of interface for data access and retrieval (manual, automatic)
	A1d. Technique to translate raw data into model parameter values (e.g., arithmetic conversion, Bayesian estimation)

Credibility and validity of model assumptions (A2)	For each of the reviewed PI models, examine assumptions concerning contact probability and frequency of new infection updates

Represent human behavior (A3)	For each of the PI models:
	- identify the human behavioral aspects addressed,
	- examine data support using criteria A1a through A1d, and
	- assess the reasons for inadequacy of human behavioral considerations

Accessibility and scalability (A4, A5)	For each PI model, examine:
	- if the model software is available to general public (open source or closed source code),
	- presence of end user support (user manuals, e-mail/phone technical support),
	- information on the number of replicates needed for valid output,
	- information on the running time,
	- information on the ways to manage the computational load for implementing large-scale scenarios (e.g., the use of distributed and parallel computing),
	- use of replicate minimization techniques, and
	- type of interface for data access and retrieval (A1c), and data translation (A1d)

#### Credibility and validity of model assumptions (A2)

Public health practitioners have emphasized the need for models with credible and valid assumptions [1]. Credibility and validity of model assumptions generally refer to how closely the assumptions represent reality. However, for modeling purposes, assumptions are often made to balance data needs, analytical tractability, and computational feasibility of the models with their ability to support timely and correct decisions [5]. Making strong assumptions may produce results that are timely but with limited or no decision support value. On the other hand, relaxing the simplifying assumptions to the point of analytical intractability or computational infeasibility may seriously compromise the fundamental purpose of the models.

Every model is comprised of multitudes of assumptions pertaining to contact dynamics, transmission and infection processes, spatial and temporal considerations, demographics, mobility mode(s), and stochasticity of parameters. Credibility and validity of these assumptions largely depend on how well they support the decision objectives of the models. For example, if a model objective is to test a household isolation strategy (allowing sick individuals to be isolated at home, in a separate room), the model assumptions must allow tracking of all the individuals within the household (primary caregivers and others) so that the contact among the household members can be assigned and possible spread of infection within the household can be assessed. This idea is further discussed in the results section through an analysis of some of the model assumptions regarding *contact probability *and *frequency of new infection updates *that were made in two of the commonly referenced PI models in the pandemic literature [14,15].

#### Ability to represent human behavior (A3)

It has been observed in [1] that the existing PI models fall short of capturing relevant aspects of human behavior. This observation naturally evokes the following questions. What are the relevant behavioral aspects that must be considered in PI models? Are there scientific evidences that establish the relative importance of these aspects? What temporal consistency is required for data support of the aspects of human behavior?

The third row of Table 2 summarizes our plan to examine how the existing models capture human behavior. For each reviewed PI model, we first identify the behavioral aspects that were considered, and then for each aspect we examine the source and the age of data used, the type of interface used for data access and retrieval, and the technique used for translating data into model parameter values (A1 a-d). We also attempt to answer the questions raised above, with a particular focus on determining what enhancements can be done to better represent human behavior in PI models.

#### Accessibility and scalability (A4, A5)

Public health practitioners have indicated the need for openly available models and population specific data that can be downloaded and synthesized using personal computers [1]. While the ability to access the models is essential for end users, executing the PI models on personal computers, in most cases, may not be feasible due to the computational complexities of the models. Some of the existing models feature highly granular description of disease spread dynamics and mitigation via consideration of scenarios involving millions of individuals and refined time scales. While such details might increase credibility and validity of the models, this can also result in a substantial computational burden, sometimes, beyond the capabilities of personal computers.

There are several factors which contribute to the computational burden of the PI models, the primary of which is the population size. Higher population size of the affected region requires larger datasets to be accessed, retrieved, and downloaded to populate the models. Other critical issues that add to the computational burden are: data interface with a limited bandwidth, the frequency of updating of data during a pandemic progress, pre-processing (filtering and quality assurance) requirement for raw data, and the need for data translation into parameter values using methods, like maximum likelihood estimation and other arithmetic conversions.

The choice of the PI model itself can also have a significant influence on the computational burden. For example, differential equation (DE) models divide population members into compartments, where in each compartment every member makes the same number of contacts (homogeneous mixing) and a contact can be any member in the compartment (perfect mixing). In contrast, agent-based (AB) models track each individual of the population where an individual contacts only the members in his/her relationship network (e.g., neighbors, co-workers, household members, etc.) [16]. The refined traceability of individual members offered by AB models increases the usage of computational resources. Further increases in the computational needs are brought on by the need for running multiple replicates of the models and generating reliable output summaries.

As summarized in the last row of Table 2, we examine which models have been made available to general public and whether they are offered as an open or closed source code. We also check for the documentation of model implementation as well as for existence of user support, if any. In addition, we look for the ways that researchers have attempted to address the computational feasibility of their models, including data access, retrieval and translation, model execution, and generation of model outputs.

### Paper screening criteria

**Figure 1:**
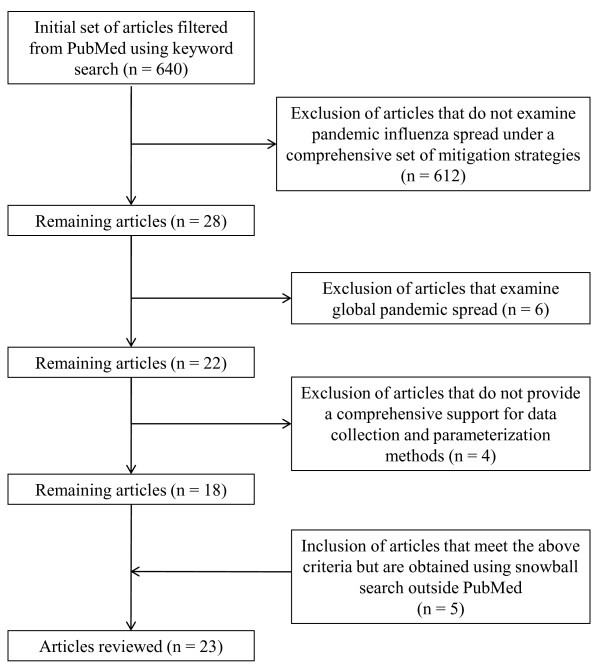
**Selection criteria for PI models for systematic review**.

The initial set of articles for our review was selected following the PRISMA reporting methodology, as applicable. We used the PubMed search engine with the keyword string "influenza" AND "pandemic" AND "model" in English language. A total of 640 papers were found which were published between 1990 and 2010. We filtered those using the following selection criteria (also depicted in Figure 1).

- Articles that evaluate one or more strategies in each of the mitigation categories: social distancing, vaccination, and antiviral application. We limited the paper (by excluding models that do not consider all three categories) to contain the scope of this review, as we examined a large body of related papers from which our selected articles drew their parameters (see additional tables).

- Articles with single-region simulation models. We defined single-region for the purpose of this review as either a country or any part thereof. Models presenting disease spread approaches without mention of any regional boundary were included, as these approaches can directly support decision makers at provincial and local levels. There exists a significant and important body of literature that is dedicated to global pandemic influenza modeling that aims at quantifying global disease spread [17-20], assessing the impact of global vaccine distribution and immunization strategies [18-20] and assessing the impact of recommended or self-initiated mobility behaviors in the global disease spread [21,22]. As these overarching aims of the global models do not directly impact operational decisions of provincial and local policy makers during an evolving pandemic, we have not included them in our final selection of articles.

- Articles that include data sources for most model parameter values and, when possible, specify the methods for parameter estimation. We included this criterion in order to evaluate models with respect to the challenge of "validity of data support." See Table 2 where we outline our evaluation plan. Clearly, models not satisfying this criterion would not support our review objectives.

Using the above filtering criteria, an additional snowball search was implemented outside PubMed, which yielded 5 additional eligible papers [14,23-26]and bringing the total number of papers reviewed to twenty-three. We grouped the twenty-three selected articles in eleven different clusters based on their model (see Table 3). The clusters are named either by the name used in the literature or by the first author name(s). For example, all three papers in the Imperial-Pitt cluster use the model introduced initially by Ferguson et al. [27]. In each cluster, to review the criteria for the design and implementation challenge (A1), we selected the article with the largest and most detailed testbed (marked in bold in Table 3). As stated earlier, credibility and validity of model assumptions (A2), were examined via two most commonly cited models in the pandemic literature [14,15]. The challenges A3-A5 were examined separately for each of the selected articles.

**Table 3:** Clustering of selected review articles based on model type

Model cluster	Selected articles for review
Imperial-Pitt	Ferguson et al. 2005 [27], **Ferguson et al. 2006 **[14], Halloran et al. 2008 [28]

Wu	**Wu et al. 2006 **[29]

Ciofi	**Ciofi et al. 2008 **[30]

Arino	**Arino et al. 2006 **[31], Arino et al. 2008 [32]

UW - LANL	Longini et al. 2004 [33], Longini et al. 2005 [34], **Germann et al. 2006 **[15], Sander et al. 2009 [35], Chao et al. 2010 [23], Halloran et al. 2008 [28]

Gojovic	**Gojovic et al. 2009 **[36]

LOKI - INFECT	**Glass et al. 2006 **[24], Davey et al. 2008 [37], Davey et al. 2008 [38], Perlroth et al. 2010 [39]

Nuno - Gumel	**Nuno et al. 2007 **[40], Gumel et al. 2008 [41]

Roberts	**Roberts et al. 2007 **[42]

Influsim	**Eichner et al. 2007 **[43]

USF	Das et al. 2008 [25], **Uribe et al. 2010 **[26]

Out of the ten model clusters presented in Table 3, eight are agent-based simulation models, while the rest are differential equation models. Also, while most of the models use purely epidemiological measures (e.g., infection attack rates and reproduction numbers) to assess the effectiveness of mitigation strategies, only a few use economic measures [26,35,39].

In our review, we examined epidemiological, demographical, and social-behavioral parameters of the pandemic models. We did not examine the parameters of the mitigation strategies as a separate category since those are functions of the epidemiological, demographical, and social-behavioral parameters. For example, the risk groups for vaccine and antiviral (which are mitigation parameters) are functions of epidemiological parameters such as susceptibility to infection and susceptibility to death, respectively. Another example is the compliance to non-pharmaceutical interventions, a mitigation strategy parameter, which can be achieved by altering the social behavioral parameters of the model.

## Results and discussion

In this section, we present the results of our review of the models that evaluate at least one strategy from each mitigation category (social distancing, vaccination and antiviral application). We also identify areas of enhancements of the simulation based PI models for operational use.

### Validity of data support

Our discussion on validity of data support includes both epidemiological and demographic data. Additional file 1: Table S1 summarizes the most common epidemiological parameters used in the selected models along with their data sources, interface for data access and retrieval, and techniques used in translating raw data into parameter values. Additional file 1: Table S2 presents information similar to above for demographic parameters.

#### Epidemiological data support

The most commonly used epidemiological parameters are reproduction number (R), illness attack rate (IAR), disease natural history parameters, and fraction of asymptomatic infected cases. In the models that we have examined, estimates of reproduction numbers have been obtained by fitting case/mortality time series data from the past pandemics into models using differential equations [44], cumulative exponential growth equations [7], and Bayesian likelihood expressions [27]. IARs have been estimated primarily using household sampling studies [33], epidemic surveys [29,45], and case time series reported for 2009 H1N1 [36,46]. The parameters of the disease natural history, which are modeled using either a continuous or phase-partitioned time scale (see Additional file 1: Table S1), have been estimated from household random sampling data [27,33,47], viral shedding profiles from experimental control studies [23,43,48,49], and case time series reported for 2009 H1N1 [36,46]. Bayesian likelihood estimation methods were used in translating 2009 case time series data [27,46]. Fraction of asymptomatic infected cases has been estimated using data sources and translation techniques similar to the ones used for natural history.

Recent phylogenetic studies on the 2009 H1N1 virus help to identify which of the above epidemiological parameters need real-time re-assessment. These studies suggest that the migratory patterns of the virus, rather than the intrinsic genomic features, are responsible for the second pandemic wave in 2009 [50,51]. Since R and IAR are affected not only by the genomic features but also by the migratory patterns of the virus, a close monitoring of these parameters throughout the pandemic spread is essential. Real-time monitoring of parameters describing disease natural history and fraction of asymptomatic cases is generally not necessary since they are mostly dependent on the intrinsic genomic features of the virus. These parameters can be estimated when a viral evolution is confirmed through laboratory surveillance. Estimation methods may include surveys (e.g., household surveys of members of index cases [52,53]) and laboratory experiments that inoculate pandemic strains into human volunteers [54].

#### Areas of enhancement

Current pandemic research literature shows the existence of estimation methodologies for IAR and R that can be readily used provided that raw data is available [46]. There exist several estimators for R (Wallinga et al. [55,56], Fraser [57], White and Pagano [58], Bettencourt et al. [59], and Cauchemez et al. [60]). These estimates have been derived from different underlying infection transmission models (e.g., differential equations, time since infection and directed network). With different underlying transmission models, the estimators consider data from different perspectives, thereby yield different values for R at a certain time t. For example, Fraser [57] proposes an instantaneous R that observes how past case incidence data (e.g., in time points t-1, t-2, t-3) contribute to the present incidence at time t. In contrast, Wallinga et al. [55,56] and Cauchemez et al. [60] propose estimators that observe how the future incidences (e.g., t + 1, t + 2, t + 3) are contributed by a case at time t. White and Pagano [58] considers an estimator that can be called a running estimate of the instantaneous reproduction number.

Further extensions of the above methods have been developed to accommodate more realistic assumptions. Bettencourt extended its R estimator to account for multiple introductions from a reservoir [59]. The Wallinga estimator was extended by Cowling [61] to allow for reporting delays and repeated importations, and by Glass [62] to allow for heterogeneities among age groups (e.g., adults and children). The Fraser estimator was extended by Nishiura [63] to allow the estimation of the reproduction number for a specific age class given infection by another age class.

The above methods for real-time estimation of R are difficult to implement in the initial and evolving stages of a pandemic given the present status of the surveillance systems. At provincial and local levels, surveillance systems are passive as they mostly collect data from infected cases who are seeking healthcare [64]. With passive surveillance, only a fraction of symptomatic cases are detected with a probable time delay from the onset of symptoms. Once the symptomatic cases seek healthcare and are reported to the surveillance system, the healthcare providers selectively submit specimens to the public health laboratories (PHL) for confirmatory testing. During the H1N1 pandemic in 2009, in regions with high incidence rates, the daily testing capacities of the PHL were far exceeded by the number of specimens received. In these PHL, the existing first-come-first-serve testing policy and the manual methods for receiving and processing the specimens further delayed the pace of publication of confirmed cases. The time series of the laboratory confirmed cases likely have been higher due to the increased specimen submission resulting from the behavioral response (fear) of both the susceptible population and the healthcare providers after the pandemic declaration [65]. Similarly, time series of the confirmed cases likely have been lower at the later stages of the pandemic as federal agencies advocated to refrain from specimen submission [66].

The present status of the surveillance systems calls for the models to account for: the underreporting rates, the delay between onset of symptoms and infection reporting, and the fear factor. In addition, we believe that it is necessary to develop and analyze the cost of strategies to implement active surveillance and reduce the delays in the confirmatory testing of the specimens. In our opinion, the above enhancement can be achieved by developing methods for statistical sampling and testing of specimens in the PHL. In addition, new scheduling protocols will have to be developed for testing the specimens, given the limited laboratory testing resources, in order to better assess the epidemiological parameters of an outbreak. With better sampling and scheduling schemes at the PHL, alterations in the specimen submission policies during a pandemic (as experienced in the U.S. during the 2009 outbreak) may not be necessary. The above enhancements would also support a better real-time assessment of the IAR, which is also derived from case incidence data.

Our review of the selected PI models indicates that currently all of the tasks relating to access and retrieval of epidemiological data are being done manually. Techniques for translation of data into model parameter values range from relatively simple arithmetic conversions to more time-consuming methods of fitting mathematical and statistical models (see Additional file 1: Table S1). There exist recent mechanisms to estimate incidence curves in real-time by using web-based questionnaires from symptomatic volunteers [67], Google and Yahoo search queries [68,69] and Tweeter messages [70] and have supported influenza preparedness in several European countries and the U.S. [67,69]. If real-time incidence estimates are to be translated into PI models parameters, complex translation techniques might delay execution of the model. We believe that model developers should consider building (semi)automatic interfaces for epidemiological data access and retrieval and develop translation algorithms that can balance the run time and accuracy.

#### Demographic data support

Additional file 1: Table S2 shows the most common demographic parameters that are used in the selected models. The parameters are population size/density, distribution of household size, peer-group size, age, commuting travel, long-distance travel, and importation of infected cases to the modeled region. Estimation of these parameters has traditionally relied on comprehensive public databases, including the U.S. Census, Landscan, Italian Institute of Statistics, Census of Canada, Hong Kong survey data, UK National Statistics, National Household Travel Survey, UK department of transport, U.S. National Centre for Educational Statistics, the Italian Ministry of University and Research and the UK Department for Education and Skills. Readers are referred to Additional file 1: Table S2 for a complete list of databases and their web addresses. Our literature review shows that access and retrieval of these data are currently handled through manual procedures. Hence, there is an opportunity for developing tools to accomplish (semi)automatic data access, retrieval, and translation into model parameters whenever a new outbreak begins. It is worth noting that access to demographic information is currently limited in many countries, and therefore obtaining demographic parameters in real-time would only be possible for where information holders (censing agencies and governmental institutions) openly share the data.

The data sources supporting parameters for importation of infected cases reach beyond the modeled region requiring the regional models to couple with global importation models. This coupling is essential since the possibility of new infection arrivals may accelerate the occurrence of the pandemic peak [17]. This information on peak occurrence could significantly influence time of interventions. Some of the single region models consider a closed community with infusion of a small set of infected cases at the beginning [24,26,34]. Single region models also consider a pseudo global coupling through a constant introduction of cases per unit time [15,29]. Other single region models adopt a more detailed approach, where, for each time unit, the number of imported infections is estimated by the product of the new arrivals to the region and the probability of an import being infected. This infection probability is estimated through a global disease spread compartmental model [14,30]. The latter approach is similar to the one used by Merler [17] for seeding infections worldwide and is operationally viable due to its computational simplicity. For a more comprehensive approach to case importation and global modeling of disease spread, see [71].

### Credibility and validity of model assumptions

Recall that our objective here is to discuss how the credibility and validity of assumptions should be viewed in light of their impact on the usability of models for public health decision making. We examine the assumptions regarding *contact probability *and the *frequency of new infection updates *(e.g., daily, quarterly, hourly) in two models: the Imperial-Pitt [14] and the UW-LANL models [15]. Choice of these models was driven by their similarities (in region, mixing groups, and the infection transmission processes), and the facts that these models were cross validated by Halloran [28] and were used for developing the CDC and HHS "Community Containment Guidance for Pandemic Influenza" [3].

We first examine the assumptions that influence *contact probabilities *within different mixing groups (see Table 4). For household, the Imperial-Pitt model assumes constant contact probability while the UW-LANL model assumes that the probability varies with age (e.g., kid to kid, kid to adult). The assumption of contact probability varying with age matches reality better than assuming it to be constant [72]. However, for households with smaller living areas the variations may not be significant. Also, neither of the papers aimed at examining strategies (e.g., isolation of sick children within a house) that depended on age-based contact probability. Hence, we believe that the assumptions can be considered credible and valid. For workplaces and schools, the assumption of *75% of contacts within the group and 25% contacts outside the group*, as made in the Imperial-Pitt model, appears closer to reality than the assumption of constant probability in the UW-LANL model [72]. For community places, the Imperial-Pitt model considered proximity as a factor influencing the contact probability, which was required for implementing the strategy of providing antiviral prophylaxis to individuals within a ring of certain radius around each detected case.

**Table 4:** Factors that influence contact probabilities within mixing groups

Mixing group	Factors that influence contact probabilities	
	Imperial-Pitt (Ferguson, 2006)	UW-LANL (Germann, 2007)
Household	Contact probabilities are constant	Contact probabilities vary with age

Neighborhood	Mixing group is not considered	Contact probabilities vary with age

Workplace	75% percent of all workplace contacts occur within a workgroup of close colleagues and the remaining 25% of contacts occur outside the workgroup. Contact probabilities in both cases are constant.	Contact probabilities are constant

School (pre-school, elementary, middle, high, university)	As in workplace	Contact probabilities are constant

Community places, e.g., churches, banks, supermarkets, afterschool	Contact probability between two members varies according to proximity	Contact probabilities vary with age

We also examined the assumptions regarding the *frequency of infection updates*. The frequency of update dictates how often the infection status of the contacted individuals is evaluated. In reality, infection transmission may occur (or does not occur) whenever there is a contact event between a susceptible and an infected subject. The Imperial-Pitt and the UW-LANL models do not evaluate infection status after each contact event, since this would require consideration of refined daily schedules to determine the times of the contact events. Instead, the models evaluate infection status every six hours [14] or at the end of the day [15] by aggregating the contact events. While such simplified assumptions do not allow the determination of the exact time of infection for each susceptible, they offer a significant computational reduction. Moreover, in a real-life situation, it will be nearly impossible to determine the exact time of each infection, and hence practical mitigation (or surveillance) strategies should not rely on it.

The above analysis reveals how the nature of mitigation strategies drives the modeling assumptions and the computational burden. We therefore believe that the policymakers and the modelers should work collaboratively in developing modeling assumptions that adequately support the mitigation strategy needs. Furthermore, the issue of credibility and validity of the model assumptions should be viewed from the perspectives of the decision needs and the balance between analytical tractability and computational complexity. For example, it is unlikely that any mitigation strategy would have an element that depends of the minute by minute changes in the disease status. Hence, it might be unnecessary to consider a time scale of the order of a minute for a model and thus increase both computational and data needs.

### Represent human behavior

Contact rate is the most common social-behavioral aspect considered by the models that we have examined. In these models, except for Eichner et al. [43], the values of the contact rates were assumed due to the unavailability of reliable data required to describe the mobility and connectivity of modern human networks [24,37,38]. However, it is now possible to find "fresh" estimates of the types, frequency, and duration of human contacts either from a recent survey at the continental level [72] or from a model that derives synthetic contact information at the country level [73]. In addition, recent advances in data collection through Bluetooth enabled mobile telephones [74] and radio frequency identification (RFID) devices [75] allow better extraction of proximity patterns and social relationships. Availability of these data creates further opportunity to explore methods of access, retrieval, and translation into model parameters. Issues of data confidentiality, cost of the sensing devices, and low compliance to the activation of sensing applications might prevent the Bluetooth and RFID technologies from being effectively used in evolving pandemic outbreaks. Another possibility is the use of aggregated and anonymous network bandwidth consumption data (from network service providers) to extrapolate population distribution in different areas at different points in time [76,77].

Other social-behavioral parameters that are considered by the reviewed models include reactive withdrawal from work or school due to appearance of symptoms [27], work absenteeism to care for sick relatives or children at home due to school closure [27,36,38,43], and compliance to social distancing, vaccination, and antiviral prophylaxis [28,37]. Once again, due to the lack of data support, the values of most of these parameters were assumed and their sensitivities were studied to assess the best and worst case scenarios. Existing surveys collected during the 2009 H1N1 outbreak can be useful in quantifying the above parameters [78,79].

Recent literature has explored many additional social-behavioral aspects that were not considered in the models we reviewed. There are surveys that quantify the levels of support for school closure, follow up on sick students by the teachers [78], healthcare seeking behavior [80], perceived severity, perceived susceptibility, fear, general compliance intentions, compliance to wearing face masks, role of information, wishful thinking, fatalistic thinking, intentions to fly away, stocking, staying indoors, avoiding social contact, avoiding health care professionals, keeping children at home and staying at home, and going to work despite being advised to stay at home [81]. There are also models that assess the effect of self-initiated avoidance to a place with disease prevalence [21], voluntary vaccination and free-ride (not to vaccinate but rely on the rest of the population to keep coverage high [82]. Other recognized behaviors include refusal to vaccinate due to religious beliefs and not vaccinating due to lack of awareness [82].

We believe that there is a need for further studies to establish the relative influence of all of the above mentioned social-behavioral factors on operational models for pandemic spread and mitigation. Subsequently, the influential factors need to be analyzed to determine how relevant information about those factors should be collected (e.g., in real-time or through surveys before an outbreak), accessed, retrieved, and translated into the final model parameter values.

It is important to mention very recent efforts in improving models for assessment of relevant social behavioral components including commuting, long distance travel behavior [20,83,84], and authority recommended decline of travel to/from affected regions [22]. For operational modeling, it would be helpful to adapt the approaches used by these models in translating massive data sets (e.g., bank notes, mobile phone user trajectories, air and commuting travel networks) into model parameter values. In addition, available new methodologies to model social-behavior that adapts to evolving disease dynamics [85] should be incorporated into the operational models.

### Accessibility and scalability

With regards to accessibility and scalability of the selected models, we first attempted to determine which of the simulation models were made available to general public, either as an open or closed source code. We also checked for available documentation for model implementation and user support, if any. Most importantly, we looked into how the researchers attempted to achieve the computational feasibility of their models (see Additional file 1: Table S3).

Three of the models that make their source codes accessible to general public are Influsim [43], Ciofi [30] and FluTE [23]. Influsim is a closed source differential equation-based model with a graphical user interface (GUI) which allows the evaluation of a variety of mitigation strategies, including school closure, place closure, antiviral application to infected cases, and isolation. Ciofi is an open source model that is coupled with a differential equation model to allow for a more realistic importation of cases to a region. FluTE is an open source model, which is an updated version of the UW-LANL [34] agent-based model. The source code for FluTE is also available as a parallelized version that supports simulation of large populations on multiple processors. Among these three softwares, Influsim has a GUI, whereas Ciofi and UW-LANL are provided as a C/C++ code. Influsim's GUI seems to be more user friendly for healthcare policymakers. FluTE and Ciofi, on the other hand, offer more options for mitigation strategies, but requires the knowledge of C/C++ programming language and the communication protocols for parallelization. Other C++ models are planning to become, or are already, publicly accessible, according to the Models of Infectious Disease Agent Study (MIDAS) survey [86]. We note that the policy makers would greatly benefit if softwares like FluTE or Ciofi can be made available through a cyber-enabled computing infrastructure, such as TeraGrid [87]. This will provide the policy makers access to the program through a web based GUI without having to cope with the issues of software parallelization and equipment availability. Moreover, the policy makers will not require the skills of programming, modeling, and data integration.

The need for replicates for accurate assessment of the model output measures and the run time per replicate are major scalability issues for pandemic simulation models. Large-scale simulations of the U.S. population reported running times of up to 6 h per replicate, depending on the number of parallel threads used [23] (see Additional file 1: Table S3 for further details). It would then take a run time of one week to execute 28 replicates of only one pandemic scenario. Note that, most of the modeling approaches have reported between 100 to 1000 replicates per scenario [24,36-41], with the exception of [14,26,28,30] which implemented between 5 to 50 replicates. Clearly, it would take about one month to run 100 replicates for a single scenario involving the entire U.S. population.

While it may not be necessary to simulate the entire population of a country to address mitigation related questions, the issue of the computational burden is daunting nonetheless. We therefore believe that the modeling community should actively seek to develop innovative methodologies to reduce the computational requirements associated with obtaining reliable outputs. Minimization of running time has been recently addressed through high performance computing techniques and parallelization by some of the MIDAS models (e.g., Epifast) and other research groups (e.g., DiCon, GSAM), as reported in [86]. Minimization of replicates can be achieved by running the replicates, one more at a time, until the confidence intervals for the output variables become acceptable [14,26].

In addition to the need of minimizing running time and number of replicates, it is also necessary to develop innovative methodologies to minimize the setting up time of operational models. These methodologies should enable the user to automatically select the level of modeling detail, according to the population to mimic (see a discussion of this framework in the context of human mobility [20]), and allow the automatic calibration of the model parameters.

### Limitations of the review

There exist several simulation models of pandemic influenza that can be used at the provincial and local levels and were not treated as part of the evaluated models in this article. Their exclusion is due to their limited available documentation discussing the choice of demographic, social-behavioral or epidemiological parameter values. We mention and discuss their relevant features in this manuscript, whenever applicable. For information about the additional models, the reader is referred to [86,88,89]. There also exist a body of literature evaluating less than three types of mitigation strategies that were not considered as part of the review, as we discussed in the methods section. This literature is valuable is providing insights about reproduction patterns [90,91], effect of cross-immunity [92], antiviral resistance [93], vaccine dosage [94,95], social-distancing [96] and public health interventions in previous pandemics [97,98].

## Conclusions

Though the literature on pandemic models is rich and contains analysis and results that are valuable for public health preparedness, policy makers have raised several questions regarding practical use of these models. The questions are as follows. Is the data support adequate and valid? How credible and valid are the model assumptions? Is human behavior represented appropriately in these models? How accessible and scalable are these models? This review paper attempts to determine to what extent the current literature addresses the above questions at provincial and local levels, and what the areas of possible enhancements are. The findings with regards to the areas of enhancements are summarized below.

Enhance the following: availability of real-time epidemiological data; access and retrieval of demographical and epidemiological data; translation of data into model parameter values.

We analyzed the most common epidemiological and demographical parameters that are used in pandemic models, and discussed the need for adequate updating of these parameters during an outbreak. As regards the epidemiological parameters, we have noted the need to obtain prompt and reliable estimates for the IAR and R, which we believe can be obtained by enhancing protocols for expedited and representative specimen collection and testing. During a pandemic, the estimates for IAR and R should also be obtained as often as possible to update simulation models. For the disease natural history and the fraction of asymptomatic cases, estimation should occur every time viral evolution is confirmed by the public health laboratories. For periodic updating of the simulation models, there is a need to develop interfaces for (semi)automatic data access and retrieval. Algorithms for translating data into model parameters should not delay model execution and decision making. Demographic data are generally available. But most of the models that we examined are not capable of performing (semi)automatic access, retrieval, and translation of demographic data into model parameter values.

Examine validity of modeling assumptions from the point of view of the decisions that are supported by the model.

By referring to two of the most commonly cited pandemic preparedness models [15,27], we discussed how simplifying model assumptions are made to reduce computational burden, as long as the assumptions do not interfere with the performance evaluation of the mitigation strategies. Some mitigation strategies require more realistic model assumptions (e.g., location based antiviral prophylaxis would require models that track geographic coordinates of individuals so that those within a radius of an infected individual can be identified). Whereas other mitigation strategies might be well supported by coarser models (e.g.,"antiviral prophylaxis for household members") would require models that track household membership). Therefore, whenever validity of the modeling assumptions is examined, the criteria chosen for the examination should depend on the decisions supported by the model.

Incorporate the following: a broader spectrum of social behavioral aspects; updated information for contact patterns; new methodologies for collection of human mobility data.

Some of the social behavioral factors that have been considered in the examined models are social distancing and vaccination compliance, natural withdraw from work when symptoms appear, and work absenteeism to care for sick family members. Although some of the examined models attempt to capture social-behavioral issues, it appears that they lack adequate consideration of many other factors (e.g., voluntary vaccination, voluntary avoidance to travel to affected regions). Hence, there is a need for research studies or expert opinion analysis to identify which social-behavioral factors are significant for disease spread. It is also essential to determine how the social behavioral data should be collected (in real-time or through surveys), archived for easy access, retrieved, and translated into model parameters. In addition, operational models for pandemic spread and mitigation should reflect the state of the art in data for the contact parameters and integrate recent methodologies for collection of human mobility data.

Enhance computational efficiency of the solution algorithms.

Our review indicates that some of the models have reached a reasonable running time of up to 6 h per replicate for a large region, such as the entire USA [14,23]. However, operational models need also to be set up and replicated in real-time, and methodologies addressing these two issues are needed. We have also discussed the question whether the public health decision makers should be burdened with the task of downloading and running models using local computers (laptops). This task can be far more complex than how it is perceived by the public health decision makers. We believe that models should be housed in a cyber computing environment with an easy user interface for the decision makers.

## Competing interests

The authors declare that they have no competing interests.

## Authors' contributions

DP conducted the systematic review and analysis of the models. TD and AS guided DP and AU in designing the conceptual framework for the review. All three jointly wrote the manuscript. RI and SM provided public health expert opinion on the conceptual framework and also reviewed the manuscript. All authors read and approved the final manuscript.

## Pre-publication history

The pre-publication history for this paper can be accessed here:

http://www.biomedcentral.com/1471-2458/12/251/prepub

## Supplementary Material

Additional file 1 **Additional file 1: Table S1 Epidemiological parameters in models for pandemic influenza preparedness**. The excel sheet "Additional file1: Table S1" shows the epidemiological parameters most commonly used in the models for pandemic influenza, the parameter data sources, and the means for access, retrieval and translation. Additional file 1: Table S2 Demographic parameters in models for pandemic influenza preparedness. The excel sheet "Additional file 1: Table S2" shows the demographic parameters most commonly used in the models for pandemic influenza, the parameter data sources, and the means for access, retrieval and translation. Additional file 1: Table S3 Accessibility and scalability features investigated in the models. The excel sheet "Additional file 3" shows the different models examined, together with their type of public access, number and running time per replicate, and techniques to manage computational burden. Click here for file
